# Cytogenetic relationships among *Citrullus* species in comparison with some genera of the tribe Benincaseae (Cucurbitaceae) as inferred from rDNA distribution patterns

**DOI:** 10.1186/s12862-016-0656-6

**Published:** 2016-04-18

**Authors:** Kun-Peng Li, Yun-Xiang Wu, Hong Zhao, Yan Wang, Xing-Ming Lü, Ji-Ming Wang, Yong Xu, Zong-Yun Li, Yong-Hua Han

**Affiliations:** Institute of Integrative Plant Biology, School of Life Sciences, Jiangsu Normal University, Xuzhou, 221116 China; National Engineering Research Center for Vegetables, Beijing Academy of Agriculture and Forestry Sciences, Key Laboratory of Biology and Genetic Improvement of Horticultural Crops (North China), Beijing, 100097 China; Zhengzhou Fruit Research Institute, Chinese Academy of Agricultural Sciences, Zhengzhou, 450009 China

**Keywords:** rDNA, Oligonucleotides probes, *Citrullus* species, Cytogenetic relationship, Fluorescence in situ hybridization

## Abstract

**Background:**

Comparative mapping of 5S and 45S rDNA by fluorescent in situ hybridization (FISH) technique is an excellent tool to determine cytogenetic relationships among closely related species.

**Results:**

In this study, the number and position of 5S and 45S rDNA loci in all *Citrullus* species and subspecies were determined. The cultivated watermelon (*C. lanatus* subsp. *vulgaris*)*, C. lanatus* subsp. *mucosospermus, C. colocynthis* and *C. naudinianus* (or *Acanthosicyos naudinianus*) had two 45S rDNA loci and one 5S rDNA locus which was located syntenic to one of the 45S rDNA loci. *C. ecirrhosus* and *C. lanatus* subsp. *lanatus* had one 45S rDNA locus and two 5S rDNA loci, each located on a different chromosome. *C. rehmii* had one 5S and one 45S rDNA locus positioned on different chromosomes. The distribution of 5S and 45S rDNA in several species belonging to other genera in Benincaseae tribe was also investigated. The distribution pattern of rDNAs showed a great difference among these species.

**Conclusions:**

The present study confirmed evolutionary closeness among cultivated watermelon (*C. lanatus* subsp. *vulgaris*), *C. lanatus* subsp. *mucosospermus* and *C. colocynthis*. Our result also supported that *C. lanatus* subsp. *lanatus* was not a wild form of the cultivated watermelon instead was a separate crop species. In addition, present cytogenetic analysis suggested that *A. naudinianus* was more closely related to *Cucumis* than to *Citrullus* or *Acanthosicyos*, but with a unique position and may be a link bridge between the *Citrullus* and the *Cucumis.*

## Background

The genus *Citrullus* belongs to the Benincaseae tribe of the Cucurbitaceae family [1]. There are four or five species in the *Citrullus,* which are *Citrullus lanatus* (Thunb.) Matsum. & Nakai, *C. colocynthis* (L.) Schrad., *C. eccirrhosus* Cogn., *C. rehmii* de Winter and *C. naudinianus* (Sond.) Hook.f. [1, 2]. *C. naudinianus* is so different from the other *Citrullus* species in gross morphology that it is placed in the genus *Acanthosicyos* (*A. naudinianus*) by Jeffrey [3]. *C. lanatus* includes three subspecies of *C. lanatus* subsp. *vulgaris* (cultivated watermelon), *C. lanatus* subsp. *mucosospermus* and *C. lanatus* subsp. *lanatus*. Both *C. lanatus* subsp. *vulgaris* and *C. lanatus* subsp. *mucosospermus* have been classified as *C. lanatus* var. *lanatus*. The *C. lanatus* subsp. *lanatus* is named as *C. lanatus* var. *citroides*, which are the synonyms with *C. caffer* and *C. amarus* [1, 3]. However, the latest study reveals that *C. lanatus* is not a biological species since *C. lanatus* subsp. *lanatus* is more closely to *C. ecirrhosus* than it is to subspecies of *C. lanatus* subsp. *vulgaris* and *C. lanatus* subsp. *mucosospermus* based on molecular phylogenetic study of the genus *Citrullus* [4]. Moreover, Chomicki and Renner [4] think that three subspecies of ‘*Citrullus lanatus*’ are unrelated species and there are seven species in *Citrullus* including *C. naudinianus.*

Although various analyses have been carried out aiming to establish phylogenetic relationships among different *Citrullus* species, the conclusions are inconsistent and the progenitor of cultivated watermelon has not yet been determined [5–11]. Cytological [12] and cross-compatibility [13] observations favored *C. colocynthis* as the ancestor of cultivated watermelon. Several studies established that *C. lanatus* var. *citroides* was phylogenetically sister to cultivated watermelon [3, 14–16]. However, *C. ecirrhosus* has been found to have closer relative relationship with cultivated watermelon based on sequencing analysis of cpDNA regions [17, 18]. Jarret and Newman [9] proposed that C. rehmii might be the ancestral to cultivated watermelon by the analysis of internal transcribed spacer sequence heterogeneity.

Mapping of 5S and 45S rDNA by fluorescent in situ hybridization (FISH) technique is an excellent tool to determine phylogenetic relationships because their map positions can reveal similarities and differences between chromosomes of related species [19–21]. To date, the position and number of rDNA loci have been determined in more than 1000 plant species with FISH [22]. These studies showed that the number and position of the 5S and 45S rDNA were usually characteristic of a given species, genus or group [23] and 5S and 45S rDNA tended to occupy similar chromosomes and positions in closely related species [24–28]. The chromosomal localization of 5S and 45S rDNA loci has been reported using the FISH technique in three *C. lanatus* subspecies [29]. The study [29] showed there were one 5S locus and two 45S rDNA loci on chromosomes 4 and 8 and the 5S rDNA locus was located syntenic to one of the 45S rDNA loci on chromosome 8 in *C. lanatus* subsp. *vulgaris* and *C. lanatus* subsp. *mucosospermus*. Whereas *C. lanatus* subsp. *lanatus* contained one 45S locus on chromosomes 4 and two 5S rDNA loci, one 5S rDNA locus was on chromosome 8 and the other 5S rDNA locus was on chromosome 11. The rDNA distribution has also been investigated in *C. colocynthis* and *C. rehmii* besides *C. lanatus* var. *lanatus* and *C. lanatus* var. *citroides* [30]. However, the chromosomes with rDNA sites have not been identified due to the lack of chromosome identification markers in Reddy et al.’ study [30]. Recently, we successfully labeled specific chromosomes using oligonucleotides (oligos) libraries as probes in cucumber [31]. In this study, the distribution of the 5S and 45S rDNA in all *Citrullus* species including three *C. lanatus* subspecies were investigated and the chromosomes with rDNA sites were identified using synthesized watermelon oligos probes to gain cyto-evolution at the chromosome level and resolve relationships among *Citrullus* species.

## Methods

### Plant materials and chromosome preparation

The tested *Citrullus* species included watermelon cultivar 97103 (*C. lanatus* subsp. *vulgaris,* 2n = 2x =22) which a draft genome has been published [29], *C. lanatus* subsp. *mucosospermus* (PI 270144, 2n = 2x =22), *C. lanatus* subsp. *lanatus* (*C. amarus*, PI 500335, 2n = 2x =22), *C. colocynthis* (PI 386021, 2n = 2x =22), *C. ecirrhosus* (PI 632751, 2n = 2x =22), *C. rehmii* (Grif 16376, 2n = 2x =22) and *A. naudinianus* (PI 596690, PI 671961 and Grif 14021, 2n = 2x =24) in this study. In order to learn about the rDNA distribution information in more species closely related with *Citrullus* species, several species belonging to other genera in Benincaseae tribe were also analyzed, including *Cucumis sativus* inbred line 9930 (2n = 2x =14), *Cucumis metuliferus* (PI 482465, 2n = 2x =24), *Lagenaria siceraria* (PI 379367, 2n = 2x = 22), *Zehneria mariothii* (PI 596649, 2n = 2x = 24), *Melothria pendula* (Grif 16574, 2n = 2x = 24), *Benincasa fistulosa* (PI 449332, 2n = 2x = 24), *Coccinia sessilifolia* (PI 596663, 2n = 2x = 24) and *Diplocyclos palmatus* (PI 618818, 2n = 2x = 24). Seeds of all PI and Grif lines were obtained from the U.S. National Plant Germplasm System (Ames, IA). The seeds of watermelon cultivar 97103 were obtained from National Engineering Research Center for Vegetables in Beijing Academy of Agriculture and Forestry Sciences. The seeds of *C. sativus* inbred line 9930 were obtained from Institute of Vegetables and Flowers in Chinese Academy of Agricultural Sciences. Root tips were harvested from germinated seeds, pretreated in 0.002 M 8-hydroxyquinoline at room temperature for 2 h to accumulate metaphase cells, and fixed in methanol:glacial acetic acid (3:1). After washing with water, root tips were macerated in 2 % cellulose and 1 % pectolyase at 37 °C for 2.5 h. Finally, the treated root tips were squashed and dried in flame. The slides were kept in −20 °C freezer for FISH.

### rDNA probes and oligo probes

The plasmids of 5S and 45S rDNA which were cloned in the vector pUC18 were kindly provided by K. Arumuganthan, University of Nebraska. The 5S and 45S rDNA were labeled with digoxigenin-dUTP and biotin-dUTP via nick translation, respectively. Two oligos libraries were developed to identify cultivated watermelon chromosomes 4, 8 and 11 with rDNA sites reported by Guo et al. [29]. Library 1 contained 10000 oligos from watermelon chromosomes 4 and 8 at the densities of 2–3 oligos per kilobases, respectively. Library 2 contained 10000 oligos from watermelon chromosomes 8 and 11 at the densities of 2–3 oligos per kilobases, respectively. The region spanned by library 2 was located adjacently the region spanned by library 1 on chromosome 8. Thus, the chromosome with overlapping signals must be chromosome 8 and the others are either 4 or 11. Two oligos libraries were also developed to identify melon chromosomes 8, 10 and 12. Library 1 contained 10000 oligos from melon chromosomes 8 and 10 at the densities of 2–3 oligos per kilobases, respectively. Library 2 contained 10000 oligos from melon chromosomes 10 and 12 at the densities of 2–3 oligos per kilobases, respectively. The region spanned by library 2 was located adjacently the region spanned by library 1 on chromosome 10. Thus, the chromosome with overlapping signals must be chromosome 10 and the others are either 8 or 12. The oligo libraries were synthesized by MYcroarray (Ann Arbor, MI). Each synthesized oligo contained 48 bp of genomic sequence, a 5′ F primer, which included the T7 RNA polymerase promoter sequence, and a 3′ R primer. Amplification and labeling of oligos libraries was the same as the protocol developed by us [31].

### Fluorescence in situ hybridization (FISH)

FISH was performed according to published protocols [32]. Firstly, slides were hybridized with oligo librariy 1 (biotin-labeled) and oligo librariy 2 (digoxigenin-labeled). After the first round of probing and image capture, coverslips were taken off carefully and wash the slides three times in 1X PBS (phosphate-buffered saline) (5 min each). The slides were then dehydrated in an ethanol series (70, 90, and 100 %, 5 min each), denatured again in 70 % formamide at 80 °C for 2 min, dehydrated in a second ethanol series, and reprobed with the 45S rDNA (biotin-labeled) and 5S rDNA (digoxigenin-labeled) sequences simultaneously. Biotin-labeled and digoxigenin-labeled probes were detected using a fluorescein isothiocyanate (FITC)-conjugated antibiotin antibody (Vector Laboratories) and a rhodamine-conjugated antidigoxigenin antibody (Roche Diagnostics), respectively. Chromosomes were counterstained with 4,6-diamidino-2-phenylindole (DAPI) in a VectaShield antifade solution (Vector Laboratories). Images were captured digitally using a CCD camera (QIMAGING, RETIGA-SRV, FAST 1394) attached to an Olympus BX63 epifluorescence microscope. Gray-scale images were captured for each color channel and then merged. Final image adjustments were done with Adobe Photoshop (Adobe Systems).

## Results

### Chromosome identification using watermelon oligos probes in *Citrullus* species and *A. naudinianus*

**Fig. 1:**
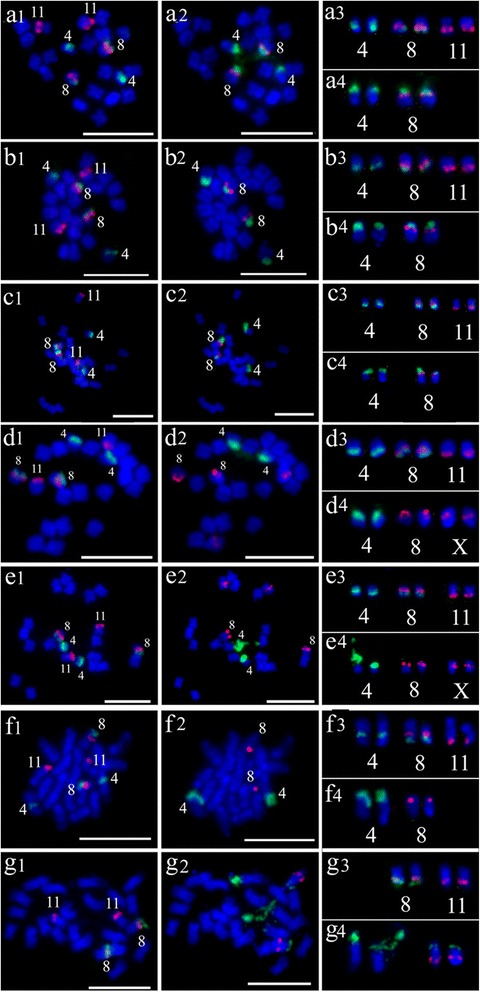
FISH of watermelon oligos probes, 5S and 45S rDNA in *Citrullus* species and *Acanthosicyos naudinianus*. **a1**-**g1** FISH oligos probes Library 1 (*green*) and Library 2 (*red*) on metaphase chromosomes of *C. lanatus* subsp. *vulgaris* (a1), *C. lanatus* subsp. *mucosospermus* (b1), *C. colocynthis* (c1), *C. lanatus* subsp. *lanatus* (d1), *C. ecirrhosus* (e1), *C. rehmii* (f1) and *A. naudinianus* (g1), respectively. **a2**-**g2** The same cells in A1-G1 were reprobed with 5S rDNA (*red*) and 45S rDNA (*green*) probes. **a3**-**g3** The chromosomes with the signals of the oligos probes Library 1 (*green*) and Library 2 (*red*) in a1-g1. **a4**-**g4** The chromosomes with the signals of 5S rDNA (*red*) and 45S rDNA (*green*) probes in a2-g2. Bars, 5 mm

It is difficult to distinguish each chromosome in watermelon because the chromosomes are relatively small and are morphologically similar. Two oligos probes were developed to identify watermelon chromosomes 4, 8 and 11. As expected, two oligo probes produced bright FISH signals on cultivated watermelon chromosomes 4, 8 and 11 (Fig. 1a1, a3). We also performed FISH on metaphase chromosomes in all *Citrullus* species including three *C. lanatus* subspecies and *A. naudinianus* in order to investigate the potential of oligo probes for cross-species hybridization. Two probes generated FISH signals with similar intensity on three pairs of chromosomes in *C. lanatus* subsp. *mucosospermus* (Fig. 1b1, b3), *C. colocynthis* (Fig. 1c1, c3), *C. lanatus* subsp. *lanatus* (Fig. 1d1, d3), *C. ecirrhosus* (Fig. 1e1, e3), *C. rehmii* (Fig. 1f1, f3), and on two pairs of chromosomes in *A. naudinianus* (Fig. 1g1, g3). The counterparts of cultivated watermelon chromosomes 4, 8 and 11 in other *Citrullus* species or subspecies and *A. naudinianus* were also designated as chromosomes 4, 8 and 11 based on the homeologous relationship with the corresponding chromosome of watermelon.

### Chromosomal distribution of 5S and 45S rDNA in *Citrullus species* and *A. naudinianus*

To determine the *distribution* of 5S and 45S rDNA in *Citrullus* species and *A. naudinianus*, following the FISH analysis of using oligos probes, the slides were washed and reprobed with 5S and 45S rDNA sequences simultaneously. The results were summarized Table 1. Consist with previous results [29], the genome of cultivated watermelon contained one 5S locus (red) and two 45S rDNA loci (green) on the chromosomes 4 and 8 (Fig. 1a2, a4). The 5S rDNA locus (red) was colocalized with the 45S rDNA loci (green) on the chromosome 8 (Fig. 1a2, a4). The number and location of 5S (red) and 45S (green) rDNA in the genomes of *C. lanatus* subsp. *mucosospermus* (Fig. 1b2, b4) and *C. colocynthis* (Fig. 1c2, c4) were identical to those in the genome of cultivated watermelon (Fig. 1a2, a4).

**Table 1:** The number of 5S and 45S rDNA loci in different species

Species	2n	No. of 5S rDNA loci (Chromosome(s) bearing 5S rDNA)	No. of 45S rDNA loci (Chromosome(s) bearing 45S rDNA)
*Citrullus lanatus* subsp. *vulgaris*	22	1 (8)	2 (4, 8)
*C. lanatus* subsp. *mucosospermus*	22	1 (8)	2 (4, 8)
*C. colocynthis*	22	1 (8)	2 (4, 8)
*C. lanatus* subsp. *lanatus*	22	2 (8, ?)	1 (4)
*C. ecirrhosus*	22	2 (8, ?)	1 (4)
*C. rehmii*	22	1 (8)	1 (4)
*Acanthosicyos naudinianus*	24	1 (?)	2 (?, ?)
*Lagenaria siceraria*	22	1	2
*Zehneria mariothii*	24	1	2
*Melothria pendula*	24	1	2
*Cucumis metuliferus*	24	1	2
*Benincasa fistulosa*	24	2	2
*Coccinia sessilifolia*	24	1	3
*Diplocyclos palmatus*	24	1	3
*Cucumis sativus*	14	1	5

The genomes of the *C. lanatus* subsp. *lanatus* (Fig. 1d2, d4) and *C. ecirrhosus* (Fig. 1e2, e4) contained two 5S loci (red) and one 45S rDNA locus (green) on the chromosomes 4. One 5S rDNA locus (with strong signals) located at the terminal end of chromosome 8, while the other 5S rDNA site (with weak signals) located intercalary near to the centromere on an unidentified chromosome x (Fig. 1d4, e4) instead chromosome 11 as that previously reported [29]. The genome of *C. rehmii* contained one 5S rDNA locus (red) on the chromosomes 8 and one 45S rDNA locus (green) on the chromosomes 4 (Fig. 1f2, f4). Like the cultivated watermelon, the genome of *A. naudinianus* also contained one 5S locus (red) and two 45S rDNA loci (green) (Fig. 1g2, g4). The 5S rDNA locus was located syntenic to one of the 45S rDNA loci. However, the 5S and 45S rDNA were far apart. Moreover, both 5S and 45S rDNA loci in *A. naudinianus* weren’t located on chromosomes 4 and 8 but on other chromosomes. In addition, *A. naudinianus* had 24 chromosomes while other *Citrullus* species had 22 chromosomes. We analyzed three *A. naudinianus* accessions (PI 596690, PI 671961 and Grif 14021) and the results were same.

### Distribution of 5S and 45S rDNA in several genera of Benincaseae tribe

**Fig. 2:**
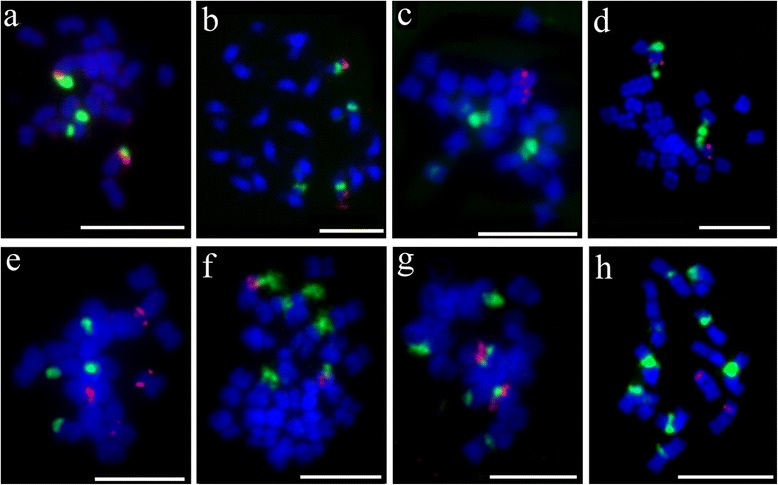
FISH of 5S rDNA (*red*) and 45S rDNA (*green*) probes in eight species from other genera in Benincaseae tribe. **a** *Lagenaria siceraria*. **b** *Zehneria mariothii*. **c** *Melothria pendula*. **d** *Cucumis metuliferus*. **e** *Benincasa fistulosa*. **f** *Coccinia sessilifolia*. **g** *Diplocyclos palmatus*. **h** *Cucumis sativus*. Bars, 5 mm

The distribution of 5S and 45S rDNA were also investigated in eight species from other genera in Benincaseae tribe, including *Lagenaria siceraria* (Fig. 2a), *Zehneria mariothii* (Fig. 2b), *Melothria pendula* (Fig. 2c), *Cucumis metuliferus* (Fig. 2d), *Benincasa fistulosa* (Fig. 2e), *Coccinia sessilifolia* (Fig. 2f), *Diplocyclos palmatus* (Fig. 2g) and *Cucumis sativus* (Fig. 2h) (Table 1). There was only one 5S locus (red) in these species except for *B. fistulosa* with two 5S loci (Fig. 2e). However, the number of 45S rDNA loci (green) showed a great difference. There were two 45S rDNA loci in *L. siceraria* (Fig. 2a)*, Z. mariothii* (Fig. 2b), *M. pendula* (Fig. 2c), *C. metuliferus* (Fig. 2d) and *B. fistulosa* (Fig. 2e), three 45S rDNA loci in *C. sessilifolia* (Fig. 2f) and *D. palmatus* (Fig. 2g), and five 45S rDNA loci in *C. sativas* (Fig. 2h), respectively. The distribution pattern of 5S and 45S rDNA and chromosome number were exactly same between *L. siceraria* (Fig. 2a) and cultivated watermelon (Fig. 1a2, a4), also between *C. sessilifolia* (Fig. 2f) and *D. palmatus* (Fig. 2g).

**Fig. 3:**
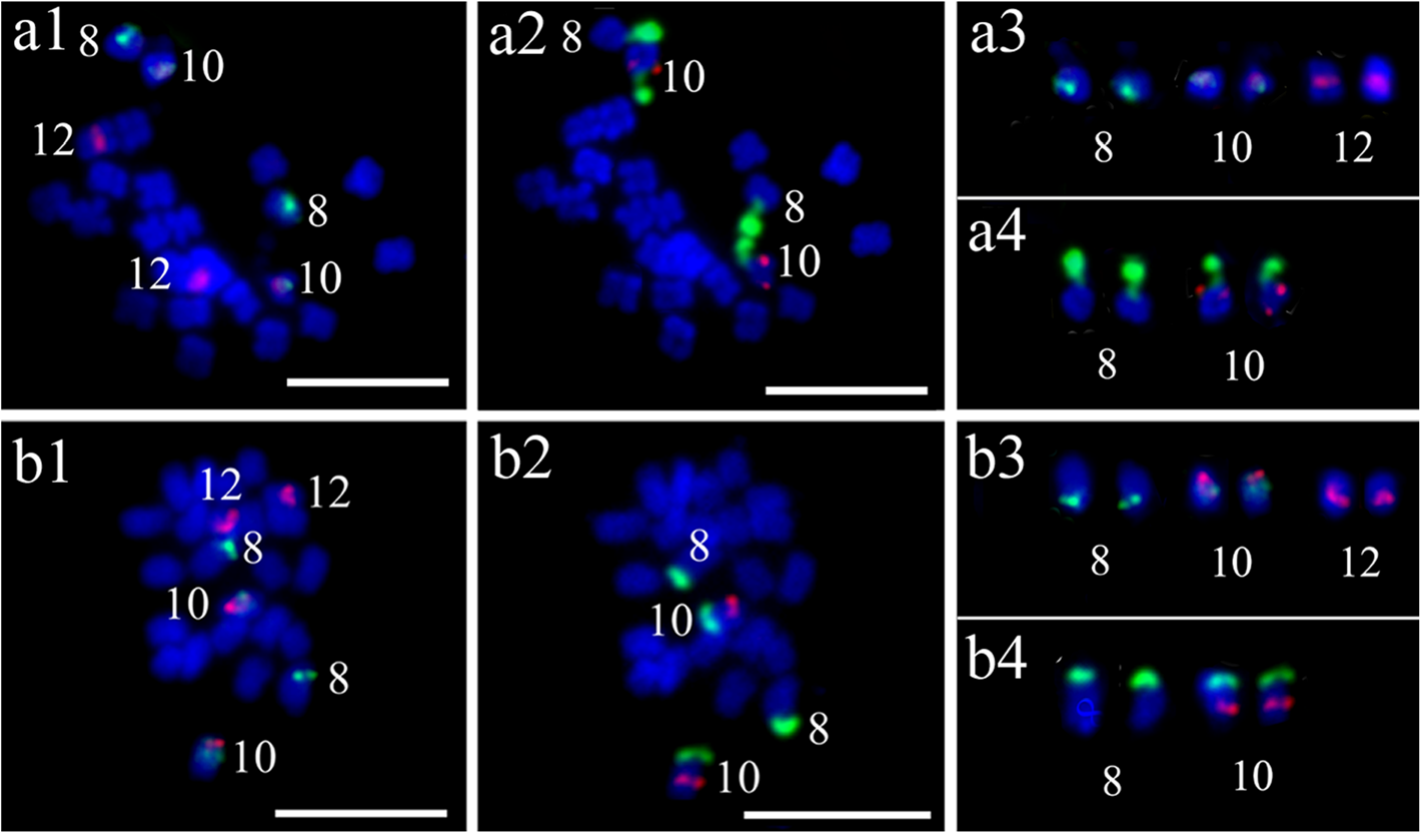
FISH of melon oligos probes, 5S and 45S rDNA in *Cucumis metuliferus* and *Acanthosicyos naudinianus*. **a1**-**b1** FISH oligos probes Library 1 (*green*) and Library 2 (*red*) on metaphase chromosomes of *C. metuliferus* (a1) and *A. naudinianus* (b1). **a2**-**b2** The same cells in a1-b1 were reprobed with 5S rDNA (*red*) and 45S rDNA (*green*) probes. **a3**-**b3** The chromosomes with the signals of the oligos probes Library 1 (*green*) and Library 2 (*red*) in a1-b1. **a4**-**b4** The chromosomes with the signals of 5S rDNA (*red*) and 45S rDNA (*green*) probes in a2-b2. Bars, 5 mm

The 5S rDNA locus was located syntenic to one of the 45S rDNA loci in *L. siceraria* (Fig. 2a), *Z. mariothii* (Fig. 2b), *C. metuliferus* (Fig. 2d), *C. sessilifolia* (Fig. 2f) and *D. palmatus* (Fig. 2g). However, only in *C. metuliferus*, the 5S and 45S rDNA were far apart (Fig. 2d). We found that *C. metuliferus* (Fig. 2d) had same number and location of 5S and 45S rDNA loci as *A. naudinianus* (Fig. 1g2, g4). To further confirm the close relationship between *C. metuliferus* and *A. naudinianus,* we performed FISH on the chromosomes of *C. metuliferus* and *A. naudinianus* using two melon oligos probes which were used to identify chromosomes 8, 10 and 12 with 5S and 45S rDNA loci in *Cucumis* species (unpublished data). Two melon probes generated distinct FISH signals on three pairs of chromosomes in *C. metuliferus* (Fig. 3a1, a3) and *A. naudinianus* (Fig. 3b1, b3). Like melon*,* the chromosomes with same oligo probes signals were also designated as chromosomes 8, 10 and 12 in *C. metuliferus* and *A. naudinianus*. Following the FISH analysis of using oligos probes, the slides were washed and reprobed with 5S and 45S rDNA sequences simultaneously. The FISH results showed that two 45S rDNA loci (green) were on the chromosomes 8 and 10 in *C. metuliferus* (Fig. 3a2, a4) and *A. naudinianus* (Fig. 3b2, b4). The 5S rDNA locus (red) was located syntenic to the 45S rDNA loci on chromosome 10 (Fig. 3a4, b4). We also tested melon oligos probes on chromosomes of other *Citrullus* species and watermelon oligos probes on chromosomes of *Cucumis* species and *L. siceraria* which was closely related to watermelon. We did not detect unambiguous signals in these species using any of the four probes (data not shown). This was consistent with our previous study that unambiguous signals weren’t detected on chromosomes of watermelon and *Cucurbita pepo* (2n = 4x = 40) using any of three cucumber oligo probes [31]. Therefore, cross-genus hybridization was not feasible using oligo probes.

## Discussion

The genus *Citrullus* belongs to the Benincaseae tribe of the Cucurbitaceae family [1]. The Benincaseae is a relatively large tribe with 204–214 species in 24 genera [33]. The phylogenetic relationships among species in this tribe were assessed by morphological [34, 35], geographical [4, 36], biochemical [37] or nucleotide data [4, 36, 38, 39]. In this study, we investigated the distribution of 5S and 45S rDNA in several genera located on the different branches of the phylogenetic tree of the tribe Benincaseae [36]. We found *L. siceraria* and cultivated watermelon had exactly same rDNA distribution. Similarly, *C. sessilifolia* had identical rDNA distribution pattern as *D. palmatus*. This was in accordance with the results based on the molecular phylogenetic studies, which revealed a close relationship between the genera *Citrullus* and *Lagenaria,* and between the genera *Coccinia* and *Diplocyclos* [4, 36].

**Fig. 4:**
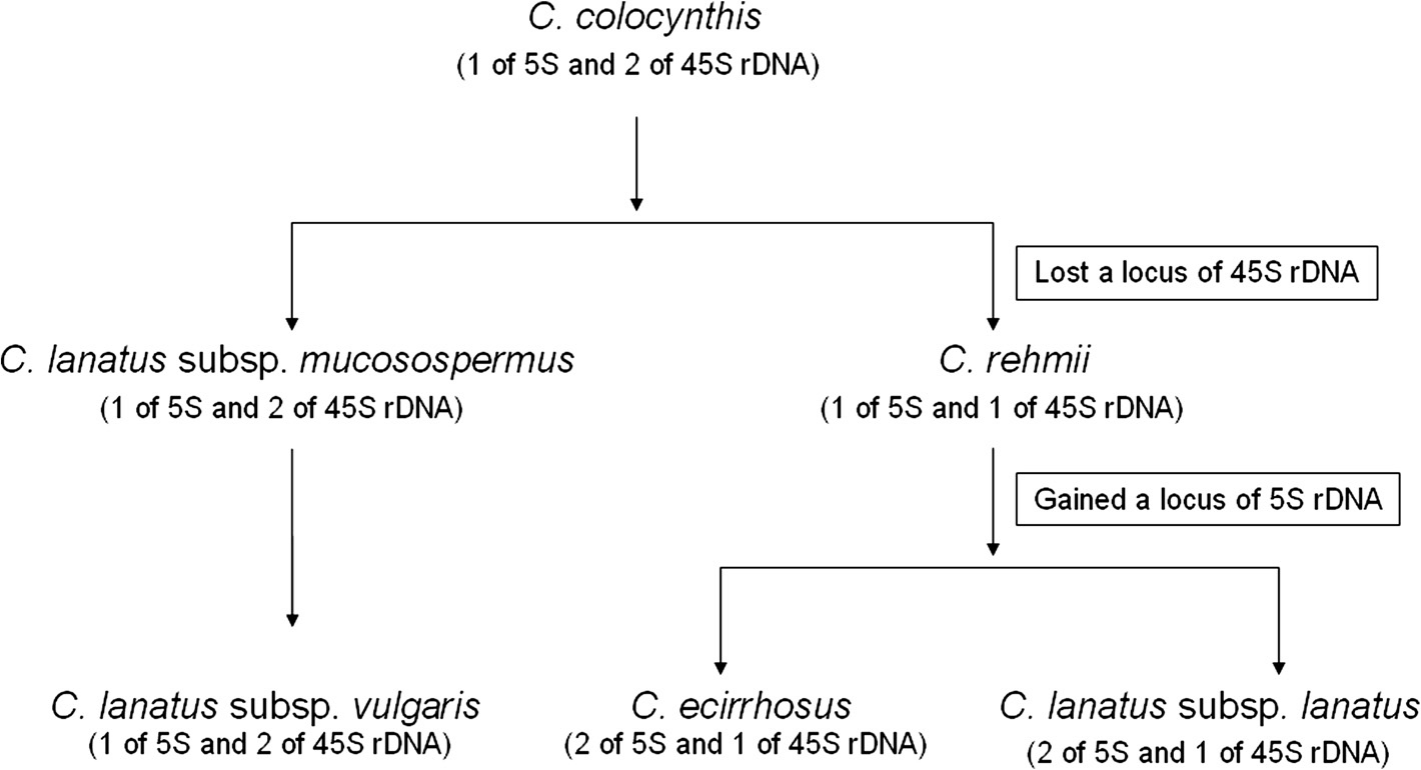
A scheme showing phylogenetic relationships among the *Citrullus* species

Consist with previous studies [29, 30], the present FISH analysis demonstrated that the rDNA gene loci showed wide differences among the *Citrullus* species. *C. lanatus* subsp. *vulgaris* (cultivated watermelon) had same number and location of 5S and 45S rDNA loci to those in *C. lanatus* subsp. *mucosospermus* and *C. colocynthis*, but differed from those in *C. lanatus* subsp. *lanatus*. Our results are concordant with assumptions that the progenitor of the cultivated watermelon might be *C. colocynthis* [12, 13] and *C. lanatus* subsp. *mucosospermus* is the recent ancestor of *C. lanatus* subsp. *vulgaris* [29]. Several reports based on nuclear and plastid data of the genus *Citrullus* also confirmed evolutionary closeness among cultivated watermelon, *C. lanatus* subsp. *mucosospermus* and *C. colocynthis*. For example, the analyses of the sequence variation at cpDNA regions also confirmed the relationship because some of the *C. lanatus* var. *lanatus* accessions shared a unique substitution at the trnE-trnT region with all *C. colocynthis* accessions [40]. Schaefer et al. [36] also confirmed that the watermelon and *C. colocynthis* evolved from a common ancestor. Our results also supported Chomicki and Renner’ findings [4], which revealed that *C. lanatus* subsp. *lanatus* was not a wild form or progenitor of the cultivated watermelon but instead was a separate crop species, domesticated independently. In addition, present cytogenetic analysis showed that *C. lanatus* subsp. *lanatus* and *C. ecirrhosus* were more closely related to each other than they are to other *Citrullus* species. Based on the current FISH analysis and previous molecular phylogenetics [4] and cytogenetic studies [29, 30], a scheme about phylogenetic relationships among the *Citrullus* species was given (Fig. 4).

One of the most striking observations from the present results was that the number, size and location of 5S and 45S rDNA signals were exactly identical between *A. naudinianus* and *C. metuliferus* in genus *Cucumis*. Moreover, two melon oligos libraries generated distinct FISH signals in *A. naudinianus.* The number and location of rDNA and oligos probes signals were completely identical between *A. naudinianus* and *C. metuliferus*. However, unambiguous signals weren’t detected when melon oligos probes hybridized to chromosomes of *Citrullus* species and watermelon oligos probes hybridized to chromosomes of *Cucumis* species. In addition, *A. naudinianus* had 24 chromosomes while other *Citrullus* species had 22 chromosomes. In the genus *Cucumis*, *C. sativus* is the only species with chromosome number 14, whereas the rest of the *Cucumis* species have a basic number 24 [41]. Our previous study also confirmed that the *A. naudinianus* had closer genetic affinity with the genus *Cucumis* than with *Citrullus* by comparative genomic in situ hybridization [42]*.* All above results suggest that *A. naudinianus* is more closely related to *Cucumis* than to *Citrullus* or *Acanthosicyos*. Interestingly, both watermelon and melon oligos probes generated distinct FISH signals on *A. naudinianus* chromosomes although cross-genus hybridization was not feasible using oligo probes in other species. Therefore, *A. naudinianus* could be a link bridge between the *Citrullus* and the *Cucumis.*

Consistent identification of individual chromosomes in a species is the foundation for successful cytogenetic research. FISH signals are reliable markers for chromosome identification. Most common FISH probes used in chromosome identification have been repetitive DNA elements or large genomic DNA clones [43–47]. Large genomic clones often contain dispersed repetitive sequences that will cause high background signal in FISH and cannot be used as chromosome-specific FISH probes [48]. The watermelon chromosomes are relatively small in size and are morphologically similar. Repetitive DNA elements were unavailable for differentiating all watermelon chromosomes. Recently, eleven BAC clones were used to differentiate eleven watermelon chromosomes and assign linkage groups to their corresponding chromosomes [49]. Furthermore, using the eleven BAC clones, 5S and 45S rDNA loci were assigned to chromosomes 4 and 8 in the genomes of cultivated watermelon and *C. lanatus* subsp. *mucosospermus*, and chromosomes 4, 8 and 11 in *C. lanatus* subsp. *lanatus* [29]. However, in present study we found that one of 5S rDNA sites was on an unidentified chromosome rather than on chromosome 11 by using oligos probes to identify chromosome 11 in *C. lanatus* subsp. *lanatus.*

## Conclusions

The present study confirmed evolutionary closeness among cultivated watermelon, *C. lanatus* subsp. *mucosospermus* and *C. colocynthis*. Our result also supported that *C. lanatus* subsp. *lanatus* was not a wild form of the watermelon instead was a separate crop species. In addition, present cytogenetic analysis suggested that *A. naudinianus* was more closely related to *Cucumis* than to *Citrullus* or *Acanthosicyos*, but with a unique position and may be a link bridge between the *Citrullus* and the *Cucumis.* In future, we will develope chromosome-specific oligos probes for identifying each watermelon chromosome. Furthermore, bulked oligo probes will be designed to cover an entire watermelon chromosome. Thus, interchromosomal rearrangements and karyotype evolution among related *Citrullus* species can be revealed in more detail through cross-species chromosome painting. The information will be useful for collection and utilization of genetic resources for cultivated watermelon improvement.

### Ethics

Not applicable.

### Consent to publish

Not applicable.

### Availability of data and materials

All data are contained within the manuscript.

## Abbreviations

FISH: fluorescent in situ hybridization
